# Cross-talk between alpha_1D_-adrenoceptors and transient receptor potential vanilloid type 1 triggers prostate cancer cell proliferation

**DOI:** 10.1186/1471-2407-14-921

**Published:** 2014-12-07

**Authors:** Maria Beatrice Morelli, Consuelo Amantini, Massimo Nabissi, Sonia Liberati, Claudio Cardinali, Valerio Farfariello, Daniele Tomassoni, Wilma Quaglia, Alessandro Piergentili, Alessandro Bonifazi, Fabio Del Bello, Matteo Santoni, Gabriele Mammana, Lucilla Servi, Alessandra Filosa, Angela Gismondi, Giorgio Santoni

**Affiliations:** School of Pharmacy, Section of Experimental Medicine, University of Camerino, Camerino, 62032 Italy; School of Biosciences and Veterinary Medicine, University of Camerino, Camerino, 62032 Italy; Department of Molecular Medicine, Sapienza University, Rome, 00161 Italy; Istituto Pasteur - Fondazione Cenci-Bolognetti, Rome, 00161 Italy; School of Pharmacy, Medicinal Chemistry Unit, University of Camerino, Camerino, 62032 Italy; Department of Medical Oncology, AOU Ospedali Riuniti, Polytechnic University of Marche, Ancona, 60126 Italy; Urology Unit, ASUR 9, Macerata, 62100 Italy; Pathology Unit, ASUR 9, Macerata, 62100 Italy

**Keywords:** α_1D_-adrenoceptors, Transient receptor potential vanilloid type 1, Noradrenaline, Proliferation, PC3 cell line, Prostate cancer

## Abstract

**Background:**

There is evidence that calcium (Ca^2+^) increases the proliferation of human advanced prostate cancer (PCa) cells but the ion channels involved are not fully understood. Here, we investigated the correlation between alpha_1D_-adrenergic receptor (alpha_1D_-AR) and the transient receptor potential vanilloid type 1 (TRPV1) expression levels in human PCa tissues and evaluated the ability of alpha_1D_-AR to cross-talk with TRPV1 in PCa cell lines.

**Methods:**

The expression of alpha_1D_-AR and TRPV1 was examined in human PCa tissues by quantitative RT-PCR and in PCa cell lines (DU145, PC3 and LNCaP) by cytofluorimetry. Moreover, alpha_1D_-AR and TRPV1 colocalization was investigated by confocal microscopy in PCa cell lines and by fluorescence microscopy in benign prostate hyperplasia (BPH) and PCa tissues. Cell proliferation was assessed by BrdU incorporation. Alpha_1D_-AR/TRPV1 knockdown was obtained using siRNA transfection. Signalling pathways were evaluated by measurement of extracellular acidification rate, Ca^2+^ flux, IP_3_ production, western blot and MTT assay.

**Results:**

The levels of the alpha_1D_-AR and TRPV1 mRNAs are increased in PCa compared to BPH specimens and a high correlation between alpha_1D_-AR and TRPV1 expression levels was found. Moreover, alpha_1D_-AR and TRPV1 are co-expressed in prostate cancer cell lines and specimens. Noradrenaline (NA) induced an alpha_1D_-AR- and TRPV1-dependent protons release and Ca^2+^ flux in PC3 cell lines; NA by triggering the activation of phospholipase C (PLC), protein kinase C (PKC) and extracellular signal-regulated kinase 1/2 (ERK1/2) pathways stimulated PC3 cell proliferation, that was completely inhibited by clopenphendioxan (WS433) and capsazepine (CPZ) combination or by alpha_1D_-AR/TRPV1 double knockdown.

**Conclusions:**

We demonstrate a cross-talk between alpha_1D_-AR and TRPV1, that is involved in the control of PC3 cell proliferation. These data strongly support for a putative novel pharmacological approach in the treatment of PCa by targeting both alpha_1D_-AR and TRPV1 channels.

**Electronic supplementary material:**

The online version of this article (doi:10.1186/1471-2407-14-921) contains supplementary material, which is available to authorized users.

## Background

Calcium (Ca^2+^) signalling is essential for regulating physiological functions such as cell proliferation and differentiation [1]. Prostate cancer is the second most lethal tumour among men, and Ca^2+^ has been shown to be essential for increased cell proliferation in advanced prostate cancer (PCa) cells [2–4]. However, the ion channel(s) involved in Ca^2+^ entry are not fully understood and the mechanism that leads to alteration of Ca^2+^ handling in PCa is still poorly defined. Understanding the factors that drive PCa towards increased cell proliferation is crucial for the development of new therapies that can prevent and/or inhibit the initiation and/or progression of PCa.

Among the transient receptor potential (TRP) channel proteins, human TRP vanilloid type 1 (TRPV1) is expressed in normal prostate epithelial cells, PCa tissues and in PC3 as well as LNCaP cells [5]. The expression of TRPV1 has been found to be significantly up-regulated in PCa compared with benign prostate hyperplasia (BPH) tissues, and the increased expression of TRPV1 correlates with increasing PCa tumour grade [6].

Alpha1-Adrenoceptors (α_1_-ARs) mediate actions of the endogenous adrenaline and noradrenaline (NA) in several target cells. On the basis of pharmacological and binding studies α_1_-ARs have been subdivided into three subtypes, namely α_1A_ (α_1a_), α_1B_ (α_1b_) and α_1D_ (α_1d_) [7]. α_1_-AR subtypes show different cellular localization: α_1B_-AR is mainly expressed on cell surface [8, 9], α_1A_-AR is evidenced on the cell surface and intracellularly [10, 11] and α_1D_-AR appears to be localized primarily perinuclearly [8, 9]. Moreover, α_1_-ARs are expressed in a variety of human tissues, including liver, kidneys, blood vessels, heart and prostate. In the human prostate α_1A_-AR and α_1B_-AR subtypes are expressed in BHP [12, 13], PCa tissues as well as in PC3 and DU145 PCa cell lines [14, 15] whereas the α_1D_-AR subtype in the PC3 cell line [16, 17].

The α_1_-ARs are G-protein-coupled receptors (GPCRs) that are linked to the heterotrimeric G-protein containing the Gαq/11/14/16 subunits. The Gαq subunit is a primary activator of phospholipase C (PLC), which promotes the cleavage of inositol 4,5-bisphosphate (PIP_2_) to yield diacylglycerol (DAG) and inositol 1,4,5-triphosphate (IP_3_). DAG and IP_3_ then promote the activation of protein kinase C (PKC). Growing evidence supports the role for α_1_-ARs in the direct mitogenic effect of catecholamines on prostate growth [18]. We previously reported the expression of the α_1B_- and α_1D_-AR subtypes in PC3 cells and the ability of NA to stimulate PC3 cell proliferation in a α_1D_-AR-dependent manner [17]. Therefore, the aim of the present study was to evaluate the correlation between α_1D_-AR and TRPV1 expression levels in patients with PCa and to demonstrate the role of TRPV1 in the regulation of NA-induced α_1D_-AR-dependent PC3 cell proliferation.

## Methods

### Prostate cancer cell line

Human prostate cancer cell lines PC3, and DU145 were purchased from American Type Culture Collection (ATCC, Rockville, MD). Cell lines were maintained in DMEM and RPMI medium (Lonza Group Ltd, Basel Switzerland), respectively, supplemented with 10% heat-inactivated fetal bovine serum (FBS, Lonza), 100 IU/ml of penicillin, 100 μg/ml of streptomycin at 37°C, 5% CO_2_ and 95% of humidity. The androgen sensitive cell line LNCaP was purchased from Istituto Zooprofilattico Sperimentale della Lombardia e dell’Emilia Romagna (IZSLER, Brescia, Italy) and maintained in RPMI supplemented as above described at 37°C, 5% CO_2_ and 95% of humidity.

### PCa tissues

Specimens (n = 37), from adult patients with high-risk clinically localized adenocarcinoma of the prostate underwent radical prostectomy at the Urology Operative Unit, ASUR 9 Macerata, were collected for qRT-PCR analysis (Additional file 1: Table S1). As control, 5 samples of BPH tissues removed by transurethral resection were used. All the specimens were embedded in paraffin and 5–7 μm-thick sections were collected on slides. Patients, giving their informed written consent, that covered the use of their tissues for research purposes, were included in the prostate cancer database of the Pathology Unit, ASUR 9. The study was approved by the Ethics committee Ospedale Civile Macerata and ASUR 9 and all procedures were conducted in accordance with the Declaration of Helsinki.

### Antibodies and reagents

The following anti-human polyclonal antibodies (Abs) were used: goat anti-TRPV1, rabbit anti-α_1D_-AR from Santa Cruz Biotechnology (Heidelberg, Germany), rabbit anti-ERK, rabbit anti-phospho p38 (anti-pp38), rabbit anti-p(Ser)-PKC substrate and rabbit anti-p38 from Cell Technology (Danvers, MA). The following anti-human mouse monoclonal Abs (mAbs) were used: anti-GAPDH (Sigma Aldrich, St. Louis, MO), anti-pERK (Cell) and anti-Bromodeoxyuridine (BrdU) fluorescein isothiocyanate (FITC)-conjugated (Prodotti Gianni, Italy). The horseradish peroxidise (HRP)-conjugated donkey anti-goat and donkey anti-mouse from Santa Cruz Biotechnology, HRP-conjugated goat anti-rabbit Ab from Cell Signaling. Purified FITC-conjugated rabbit anti-goat (RAG)IgG (EMD Chemicals, Inc. San Diego, CA), phycoerythrin (PE)-conjugated goat anti-rabbit (GARB) IgG (BD Biosciences, San Jose, CA), Alexa Fluor 488-conjugated rabbit anti-goat and Alexa Fluor 594-conjugated goat anti-rabbit Abs (Invitrogen, Carlsbad, CA) were used as secondary Abs. {2-[2-(4-Chlorobenzyloxy)phenoxy]ethyl}-[2-(2,6-dimethoxyphenoxy)ethyl]amine (clopenphendioxan, WS433, α_1D_-AR antagonist) was provided by Prof. Wilma Quaglia, School of Pharmacy, University of Camerino [17]. NA, 3-(4,5-dimethylthiazol-2-yl)-2,5-diphenyltetrazolium bromide (MTT), BrdU and dimethyl sulfoxide (DMSO, used as vehicle) from Sigma Aldrich. Chelerythrine chloride (PKC inhibitor), U73122 (PLC inhibitor), PD98059 (MEK inhibitor) and the TRPV1 antagonist, capsazepine (CPZ) [19] were purchased from Tocris Bioscience (Bristol, UK).

### Double immunofluorescence and flow cytometry

To determine the co-expression of TRPV1 and α_1D_-AR, 3×10^5^ PC3, DU145 and LNCaP cells were fixed with 4% paraformaldheyde in PBS for 10 min at room temperature, washed with permeabilizing solution (1% FBS, 0.1% saponin and 0.1% sodium azide in PBS) and stained for 30 min at 4°C first with anti-TRPV1 Ab (1:25) followed by FITC-RAG (1:40) and then with anti-α_1D_-AR (1:25) followed by PE-GARB (1:40). Normal goat and rabbit serum were used as negative control. In some experiments double immunofluorescence and FACS analysis were performed in PC3 cells double silenced for α_1D_-AR and TRPV1 genes. The percentage of positive cells determined over 10,000 events was analyzed on a FACScan cytofluorimeter (BD Bioscience) and fluorescent intensity was expressed in arbitrary units on a logarithmic scale.

### Confocal laser scanning microscopy analysis

2 × 10^5^/mL PC3 cells grown for 24 h at 37°C in poly-L-lysine coated slides, were permeabilized using 2% of paraformaldehyde with 0.5% of Triton X-100 in PBS and fixed by 4% of paraformaldehyde in PBS. After washes in PBS, cells were incubated with 3% of bovine serum albumin (BSA) and 0.1% of Tween-20 in PBS for 1 h at room temperature and then double stained with anti-TRPV1 (1:25) and anti-α_1D_-AR (1:25) Abs overnight at 4°C. Finally, samples were washed with 0.3% of Triton X-100 in PBS, incubated with Alexa Fluor 488-conjugated and Alexa Fluor 594-conjugated secondary Abs (1:100) for 1 h at 37°C and analysed with MRC600 confocal laser scanning microscope (BioRad, Hercules, CA) equipped with a Nikon (Diaphot-TMD) inverted microscope. Fluorochrome was excited with the 600 line of an argon-kripton laser. Serial optical sections were taken at 1-μm intervals through the cells. Images were processed using Jacs Paint Shop Pro (Jacs Sotfware Inc).

### Fluorescence microscopy analysis

The co-expression of α_1D_-AR and TRPV1 in tissue specimens from patients with adenocarcinoma or BHP, used as control, was evaluated by double immunofluorescence. Briefly fixed paraffin-embedded tissue slices were deparaffinized, rehydrated and washed with 0.3% Triton X-100 in PBS. After incubation with 3% of BSA and 0.3% of Triton X-100 in PBS for 1 h at room temperature, sections were first stained with anti-α_1D_-AR (1:25) Ab overnight at 4°C, washed with 0.3% of Triton X-100 in PBS and then labelled with Alexa Fluor 594-conjugated secondary Ab (1:100) for 1 h at 37°C. Subsequently, samples were washed with 0.3% of Triton X-100 in PBS and incubated with anti-TRPV1 (1:25) overnight at 4°C followed by Alexa Fluor 488-conjugated Ab (1:100) for 1 h at 37°C. Sections were analyzed using a BX51 fluorescence microscope at 10× magnification (Olympus, Milan, Italy). Merge images were obtained by using the DP controller software (Olympus).

### Western blot analysis

PC3 cells were lysed in lysis buffer (10 mM Tris, pH 7.4, 100 mM NaCl, 1 mM EDTA, 1 mM EGTA, 1 mM NaF, 20 mM Na_4_P_2_O_7_, 2 mM Na_3_VO_4_, 1% Triton X-100, 10% glycerol, 0.1% SDS, 0.5% deoxycholate, 1 mM phenylmethylsulfonylfluoride) containing protease inhibitor cocktail (SigmaAldrich) by using the Mixer Mill MM300 (Qiagen GmbH, Hilden, Germany). Lysates from PC3 cells treated for different times with NA (100 μM), WS433 (1 μM) and CPZ (1 μM) alone or in combination, were separated on 8 and 12% SDS-polyacrylamide gels, transferred and blotted with anti-pERK (1:1000) mAb followed by HRP-conjugated anti-mouse (1:1000) Ab, anti-ERK (1:1000), anti-p(Ser)-PKC substrate (1:1000), anti-pp38 (1:2000) and anti-p38 (1:2000) Abs followed by HRP-conjugated anti-rabbit (1:2000) Ab. Anti-GAPDH mAb was used as protein loading control. To verify silencing efficiency, lysates from siTRPV1 or siα_1D_-AR PC3 cells were immunoblotted with anti-TRPV1 (1:100) or anti-α_1D_-AR (1:1000) Abs followed by HRP-conjugated anti-goat (1:1000) and anti-rabbit (1:2000) Abs respectively. Immunoreactivity was detected using the LiteAblot ®PLUS (EuroClone, Milan, Italy) kits and densitometric analysis was carried out by evaluating three independent experiments by a Chemidoc using the Quantity One software (BioRad, Hèrcules, CA).

### Measurement of extracellular acidification rate (ECAR)

The eight-channel CytosensorTM microphysiometer (Molecular Devices Corp., Sunnyvale, CA, USA) was used as previously described [20] to evaluate small changes in the extracellular release of protons induced by NA in the culture medium surrounding PC3 cells. Briefly, 3 × 10^5^ PC3 cells were seeded into 12-mm capsule cups and cultured for 24 h. Then the capsule cups were loaded into the sensor chambers and the chambers were perfused with running medium (bicarbonate-free DMEM with 0.584 g/L glutamine and 2.59 g/L NaCl) at a flow rate of 100 μL /min. PC3 cells were stimulated for 30 sec with vehicle, NA (100 μM), WS433 (1 μM), CPZ (1 μM), alone or in combination, diluted into running medium and perfused through either fluid path. ECAR rate data were expressed as percentages of response respect to the baseline value.

### Gene silencing

siGENOME SMARTpools for TRPV1 (siTRPV1) and for α_1D_-AR (siα_1D_-AR) consisting of four RNA duplex targeting respectively TRPV1 and α_1D_-AR genes, and a siCONTROL non-targeting small interfering RNA (siGLO) with at least four mismatches to any human gene used as negative control were purchased from Dharmacon (Lafayette, CO). Briefly, 4×10^4^/ml PC3 cells were plated and after an overnight incubation, 20 nM of siTRPV1, siα_1D_-AR or siGLO was added to the wells, following METAFECTENE SI (Biontex Laboratories GmbH, Martinsried/Planegg, Germany) transfection protocol. Cells were harvested at day 2 post-transfection for analysis.

### Total RNA extraction and complementary DNA synthesis

Total RNA from siGLO, siTRPV1 and siα_1D_-AR PC3 cells was extracted with the RNeasy Mini Kit (Qiagen GmbH, Hilden, Germany). Total RNA from fixed paraffin-embedded tissue slices (5–7 μm-thick) was isolated by Absolutely RNA® formalin-fixed, paraffin-embedded (FFPE) kit (Stratagene, Austin, TX, USA). Five hundred ng of extracted RNA were subjected to reverse transcription using the High-Capacity cDNA Archive Kit (Life Technologies Corporation, Carlsbad, CA). One μL of the resulting cDNA products was used as template for qRT-PCR quantification.

### qRT-PCR analysis

qRT-PCR was performed using the iQ5 Multicolor Real-Time PCR Detection System (Bio-Rad). The reaction mixture contained the Syber Green Master Mix (Bio-Rad) and primer sets. Human β-actin, TRPV1 and α_1D_-AR primers designed with Primer Premier 5 (Bio-Rad, Hèrcules, CA) and purchased from SigmaAldrich. Primers sequences were: β-actin: forward 5′-ATCAGCAAGCAGGAGTATGACG -3′; reverse 5′-AAAGCCATGCCAATCTCATCTG-3′; TRPV1: forward 5′ -CTGATGGCAAGGACGACTACC-3′; reverse: 5′ -TTGACCGCAGGGAGAAGCTC-3′; α_1D_-AR: forward 5′ -GGTCGTAGCCCTGGTGGTG -3′; reverse: 5′ -CGGAGGAGAAGACAGCGTAGC -3′. Each amplification consisted of heat activation for 15 min at 95°C followed by 40 cycles at 95°C for 10 sec and 60°C for 50 sec. All samples were assayed in triplicate in the same plate and in three different experiments. Measurement of β-actin levels was used to normalize mRNA contents. TRPV1 and α_1D_-AR levels were calculated by the 2^-ΔΔCt^ method and expressed as relative fold respect to control levels.

### [Ca^2+^]_i_ measurement

3×10^6^/mL PC3 cells were washed in calcium and magnesium free PBS supplemented with 4.5 g/L of glucose used as experimental medium. Cells were resuspended in the medium supplemented with 7 μmol/L FLUO 3-AM and 1 μg/mL Pluronic F-127 (Life Technologies Corporation, Carlsbad, CA) and incubated in the dark for 30 min at 37°C, 5% CO_2_. After washing, cells were resuspended in the medium containing or not 2 mmol/L Ca^2+^ and stimulated with vehicle, NA (100 μM), WS433 (1 μM), CPZ (1 μM), alone or in combination. Fluorescence was measured by FACScan; not stimulated cells were analyzed for 2 min to establish baseline fluorescence levels.

### Proliferation assay

BrdU incorporation was determined in PC3 cells treated for 24 h with NA (100 μM), WS433 (1 μM) and CPZ (1 μM), alone or in combination and in siGLO, siTRPV1 and siα_1D_-AR PC3 cells treated with NA (100 μM) for 24 h. Cells were labelled by adding 20 μL/well of BrdU. After trypsinization and washing in PBS supplemented with 0.5% BSA and 2 mM ethylenediaminetetraacetic acid, cells were fixed for 30 min in PBS containing 30% methanol and 0.4% paraformaldheyde, permealized with PBS containing 1% paraformaldheide and 0.01% Tween-20, and then incubated for 15 min in DNAse buffer containing 500 KU/mL of DNAse (SigmaAldrich). Thereafter, cells were stained with anti-BrdU FITC-conjugated Ab (1:10) incubated for l h at room temperature and washed in PBS containing 0.5% BSA and 2 mM ethylenediaminetetraacetic acid. Samples were analyzed by a FACScan cytofluorimeter as above described.

### MTT assay

Cell growth was measured by MTT assay. 4 × 10^4^ PC3 cells/mL were plated in a 96-well microtiter plate, treated for 24 h with NA (100 μM), WS433 (1 μM) and CPZ (1 μM), alone or in combination and then incubated with 0.8 mg/ml of MTT for the last 3 h. Four replicates were used for each treatment. The supernatants were discarded and colored formazan crystals, dissolved with 100 μL/well of DMSO, were read at 570 nm wavelength by an ELISA reader (BioTek Instruments, Bad Friedrichshall, Germany). In some experiments, MTT assay was performed in PC3 cells treated for 24 h with NA (100 μM), chelerythrine (0.5 μM), U73122 (5 μM) and PD98059 (50 μM), alone or in combination. Dose response curves have been performed after 24 h treatments for chelerythrine, U73122 and PD98059 compounds (Additional file 2: Figure S1F). The highest doses that did not affect cell viability were used.

### Inositol-1,4,5-trisphosphate (IP_3_) measurement

IP_3_ was measured using the inositol-1,4,5-trisphosphate [^3^H] radioreceptor assay kit (PerkinElmer Life Sciences, Inc., Waltham, MA) [21]. Briefly, 1.5×10^5^/mL PC3 cells were treated with NA (100 μM), WS433 (1 μM) and CPZ (1 μM), alone or in combination for different times in DMEM supplemented with 1% FBS. After treatment IP_3_ was read by β scintillation counter in 5 ml of Atomlight scintillation cocktail (PerkinElmer).

### Statistical analysis

The statistical significance was determined by one-way Anova or by 2-way Anova with Bonferroni post-test. Unpaired t tests and Spearman’s rank correlation tests were performed with GraphPad Prism version 5.0 (GraphPad Software, San Diego, CA, USA). No differences were found comparing vehicle-treated with untreated PC3 cells (control).

## Results

### α_1D_-AR and TRPV1 are co-expressed in the prostate cancer cell lines

**Figure 1:**
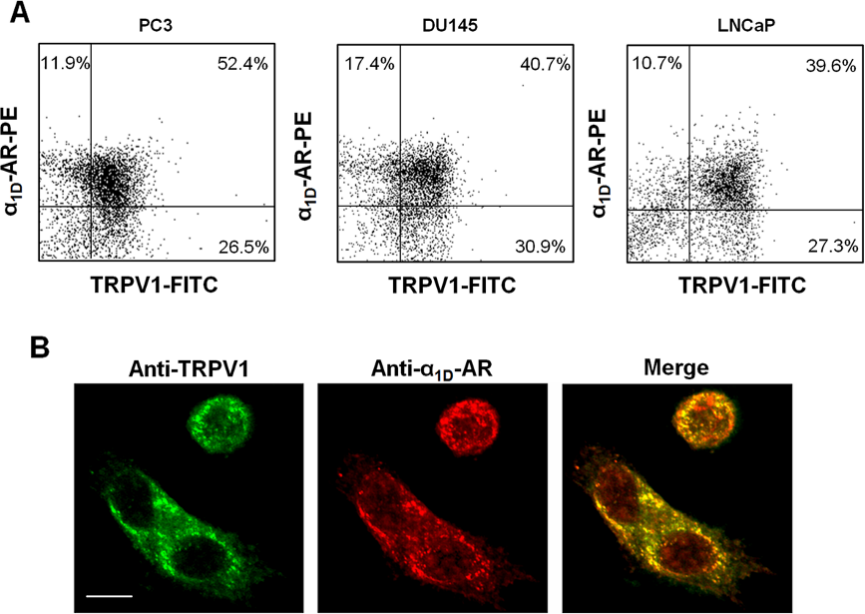
**α**_**1D**_ **-AR and TRPV1 are co-expressed and co-localized in prostate cancer cells. (A)** FACS analysis was performed in PC3, DU145 and LNCaP cells double-stained with anti-TRPV1 and anti-α_1D_-AR Abs followed by respective secondary Abs. **(B)** Confocal microscopy analysis was performed using PC3 cells grown for 24 h on poly-L-lysine coated slides, permeabilized and double-stained with anti-TRPV1 and anti-α_1D_-AR Abs, followed by Alexa Fluor 488- and Alexa Fluor 594-conjugated secondary Abs, respectively. Bar: 20 μm. Data shown are representative of one out of three separate experiments.

Previously reports have evidenced the expression of the vanilloid receptor, TRPV1 [6] and the α_1D_-AR [17] in the PC3 cell line; however, data on correlation between these receptors in PCa has not been provided so far. Therefore, by double immunofluorescence and flow cytometric analysis, we found that the α_1D_-AR and TRPV1 proteins were co-expressed in the androgens-resistant PC3 and DU145 cell lines, although at different levels. Approximately 78.9% and 64.3% of PC3 cells and 71.6% and 58.1% of DU145 expressed TRPV1 and α_1D_-AR, respectively, with 52.4% and 40.7% of them co-expressing both receptors (Figure 1A). In addition double immunofluorescence, performed in the androgens sensitive cell line LNCaP, showed that about 66.9% and 50.3% of this cell line expressed TRPV1 and α_1D_-AR, with 39.6% co-expressing both receptors, suggesting that the co-expression of α_1D_-AR and TRPV1 does not depend on the androgen sensitivity. Moreover, by confocal microscopy, we observed the co-localization of α_1D_-AR and TRPV1 primarily in the plasma membrane and in intracellular perinuclear vesicles of PC3 cells (Figure 1C).

### Co-expression of α_1D_-AR and TRPV1 in PCa tissues

**Figure 2:**
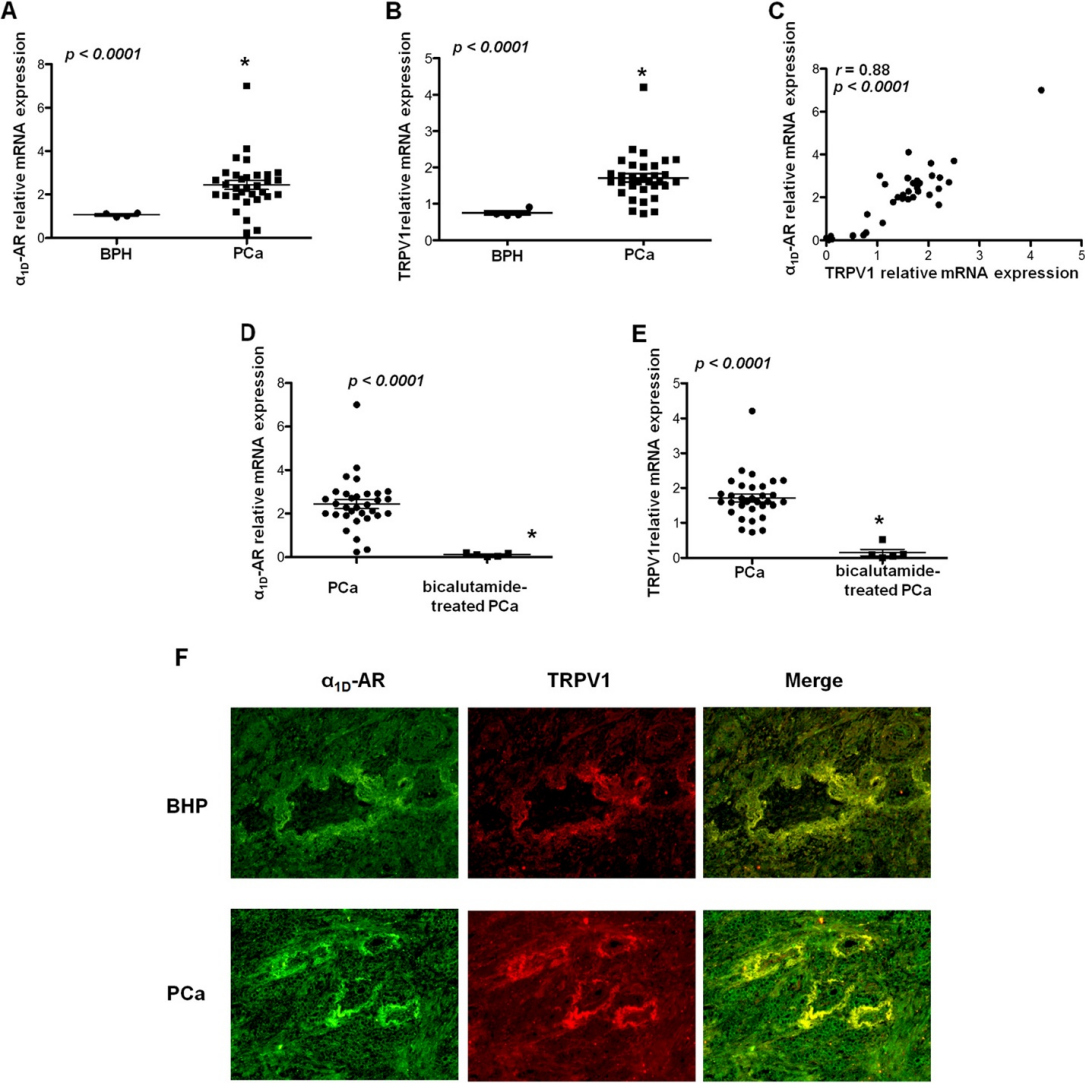
**Co-expression of** **α**_**1D**_ **-AR and TRPV1 in PCa specimens. (A-B)** Fold expression levels of α_1D_-AR and TRPV1 in PCa and BPH specimens analysed by qRT-PCR. Each point represents the expression level in a single subject. **(C)** Correlation between the α_1D_-AR and TRPV1 expression levels. Spearman’s rank correlation coefficient (r) is indicated in the graph. **(D-E)** Fold expression levels of α_1D_-AR and TRPV1 in PCa and bicalutamide-treated PCa specimens analysed by qRT-PCR. Each point represents the expression level in a single subject. The horizontal bars within the plots indicate the median percentage for each group. Significant differences between groups were identified by unpaired t tests and are indicated above the groups: *p < 0.0001. **(F)** Fluorescence microscopy analysis was performed in PCa and BHP tissues double-stained with anti-TRPV1 and anti-α_1D_-AR Abs, followed by Alexa Fluor 488- and Alexa Fluor 594-conjugated secondary Abs, respectively. Magnification: 10×. Data shown are representative of one out of three separate experiments.

The results obtained in prostate cancer cell lines were confirmed at mRNA levels in PCa specimens. Thus, we assessed the expression of α_1D_-AR mRNA in tissues from prostatectomised patients (n = 37) with advanced PCa (Additional file 1: Table S1). Using quantitative RT-PCR (qRT-PCR), we found that α_1D_-AR was over-expressed (2.44 ± 0.20) in PCa compared with BPHs (1.0 ± 0.05) (Figure 2A). As previously reported [6], we observed that TRPV1 mRNA expression significantly increased in PCa tissues (1.84 ± 0.12) compared with BPH tissues (0.61 ± 0.01) (Figure 2B); interestingly we found that TRPV1 levels highly correlated with α_1D_-AR expression (correlation index: r = 0.88; p < 0.0001) (Figure 2C). Dramatic reduction in the α_1D_-AR and TRPV1 mRNA levels was observed in PCa specimens (n = 5, pathological staging: pT3b) from patients that had received neoadjuvant androgen deprivation therapy compared with tissues from untreated PCa patients (Figure 2D and E). Moreover, by using double immunofluorescence and fluorescence microscopy analysis, we also showed that α_1D_-AR and TRPV1 proteins are co-expressed in PCa tissues and that their expression is increased in PCa compared with BPH tissues (Figure 2F).

### The cross-talk between α_1D_-AR and TRPV1 receptors

**Figure 3:**
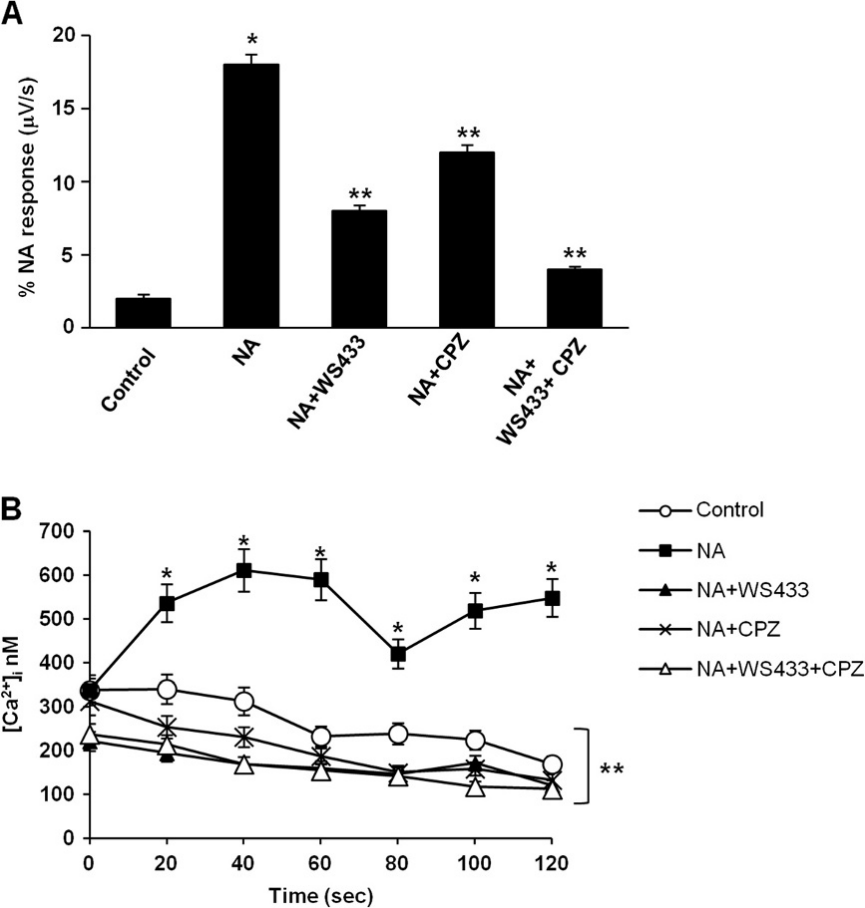
**The** α_**1D**_ **-AR and TRPV1 cross-talk in PC3 cells. (A)** The measurement of the extracellular acidification rate using an eight-channel Cytosensor TM microphysiometer was performed with PC3 cells stimulated for 30 sec with vehicle (control) or NA (100 μM) alone or in combination with WS433 (1 μM) or/and CPZ (1 μM). Data shown are the mean ± SD of three separate experiments. Statistical analysis was performed by comparing NA-treated cells with control cells (*) and NA + WS433-, NA + CPZ- and NA + WS433 + CPZ- with NA-treated cells (**), p ≤ 0.01. **(B)** The time course of [Ca^2+^]_i_ increase in Fluo-3-loaded PC3 cells vehicle-treated (control) or treated with NA alone or in combination with WS433 or/and CPZ was evaluated by FACS analysis. Data shown are the mean ± SD of three separate experiments. Statistical analysis was performed by comparing NA-treated cells with control cells (*) and NA + WS433-, NA + CPZ- and NA + WS433 + CPZ- with NA-treated cells (**), p ≤ 0.01.

The ability of NA to sensitise TRPV1 was studied in PC3 cells. First, we used a CytosensorTM microphysiometer to evaluate the μV/sec response induced by NA stimulation (100 μM) with or without α_1D_-AR antagonist, WS433, (1 μM) and TRPV1 antagonists, CPZ (1 μM). We found that protons release following NA administration, was not only α_1D_-AR- but also TRPV1-dependent as shown by the inhibitory effects induced by both WS433 (60%) and CPZ (35%). In addition, the combination of WS433 and CPZ completely inhibited the NA-induced acid activation (Figure 3A). No activation was found when using WS433 and CPZ alone respect to control PC3 cells (Additional file 2: Figure S1A). Thereafter, we followed NA-induced Ca^2+^ flux by FACS analysis in PC3 cells loaded with Fluo-3 AM. NA stimulation in Ca^2+^-containing PBS/glucose medium induced a rapid response that peaked at 40 sec after treatment with an increase in [Ca^2+^]_i_ from 300 to 650 nM (Figure 3B). Next, we addressed the contribution of both α_1D_-AR and TRPV1 components by using WS433 and CPZ. Complete abrogation of the NA-induced increase in [Ca^2+^]_i_ was observed with all the antagonists (Figure 3B). No Ca^2+^ flux was found when using WS433 and CPZ alone respect to control PC3 cells (Additional file 2: Figure S1B).

### The α_1D_-AR/TRPV1 cross-talk in response to NA activates specific signalling pathways

**Figure 4:**
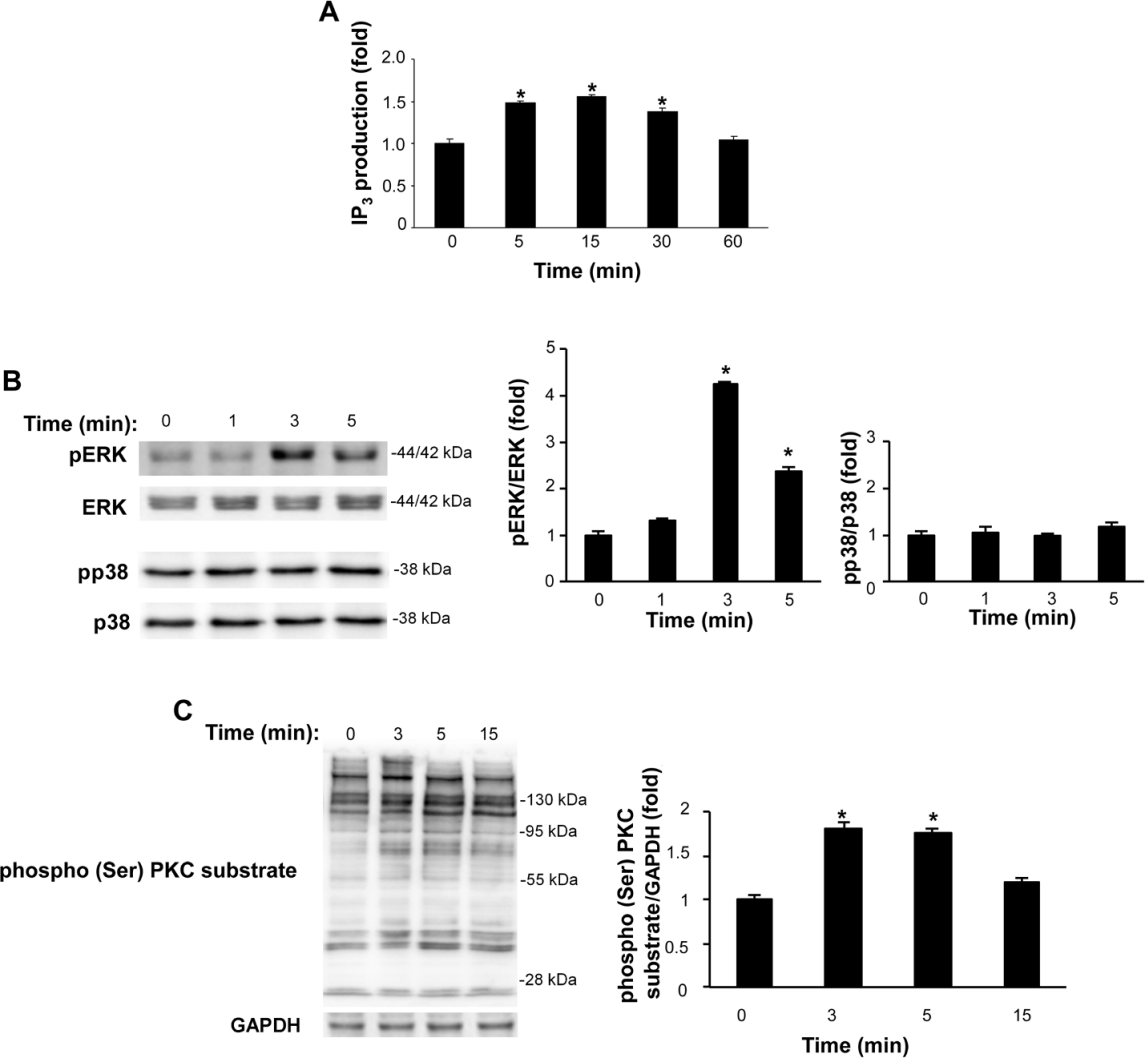
**NA induces PLC, PKC and ERK activation in PC3 cells. (A)** The measurement of IP_3_ levels using the IP_3_ [^3^H] Radioreceptor assay kit was performed in PC3 cells vehicle-treated (control) or treated for different times with NA (100 μM). Data shown are the mean ± SD of three separate experiments. Statistical analysis was performed by comparing NA-treated with control cells, *p ≤ 0.01. **(B-C)** Lysates from PC3 cells vehicle-treated (control) or treated for different times with NA (100 μM) were separated on SDS-polyacrylamide gels, transferred and then blotted with anti-pERK, anti-ERK, anti-p38, anti-pp38 and anti-phospho-(Ser) PKC substrate Abs. The GAPDH protein level was evaluated as a loading control. Data shown are representative of one out of three separate experiments. Densitometric analysis was performed evaluating three different experiments and statistical analysis was carried out comparing NA-treated with control cells, *p ≤ 0.01.

Several reports have examined the role of the PLC-PKC-MAPK signalling pathways in the catecholamine-induced α_1D_-AR-mediated proliferation of prostate cancer cells [22, 23]. Thus, we studied the effect of α_1D_-AR and TRPV1 cross-talk in the NA-induced activation of PLC, PKC and ERK signalling pathways. Time-course analysis of NA-induced PLC activation, evaluated as IP_3_ production, evidenced that NA stimulates IP_3_ production 5 min after NA treatment, remaining sustained at 30 min and declining thereafter (Figure 4A). Moreover, we found that ERK1/2 is phosphorylated at basal level and NA induces a rapid and transient increase of its phosphorylation at 3 min after treatment (Figure 4B); NA also increased the (Ser)-PKC substrate phosphorylation at 3–5 min and then return to basal level (Figure 4C); p38 was basally phosphorylated, and NA stimulation did not affect its phosphorylation state (Figure 4B).

**Figure 5:**
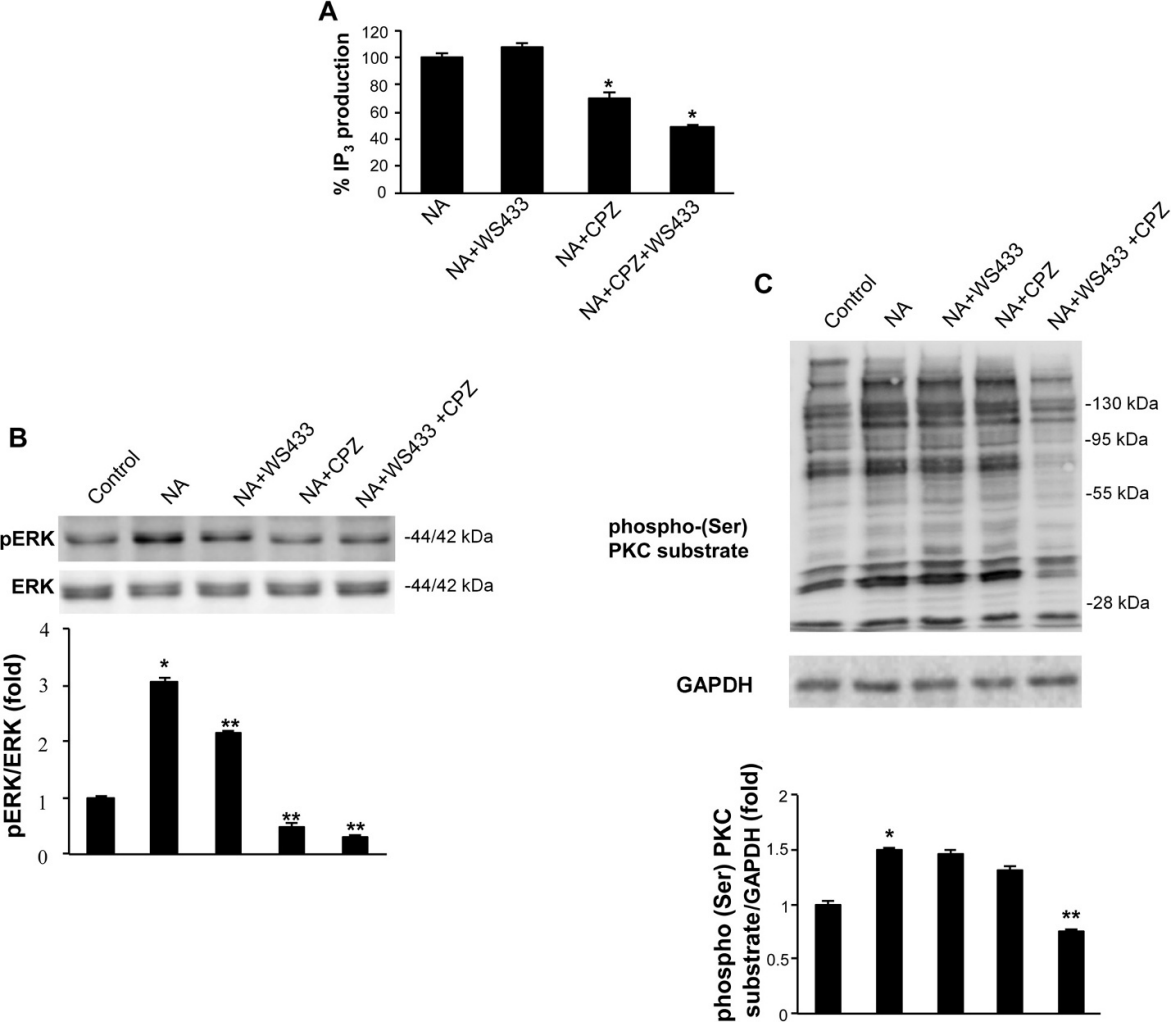
**Role of TRPV1 and** α_**1D**_ **-AR in NA-induced PLC, PKC and ERK activation. (A)** The IP_3_ levels, measured as above described, were evaluated in PC3 cells treated for 5 min with NA (100 μM), either alone or in combination with CPZ (1 μM), WS433 (1 μM) or CPZ + WS433. Data shown are the mean ± SD of three separate experiments. Statistical analysis was performed by comparing NA + WS433-, NA + CPZ- or NA + CPZ + WS433- with NA-treated cells, *p ≤ 0.01. **(B-C)** Lysates from PC3 cells vehicle-treated (control) or treated for 3 min with NA (100 μM) alone or in combination with WS433 (1 μM) and/or CPZ (1 μM), were separated on SDS-polyacrylamide gels, transferred and then blotted with anti-pERK, ERK and anti-phospho-(Ser) PKC substrate Abs. The GAPDH protein level was evaluated as a loading control. Data shown are representative of one out of three separate experiments. Densitometric analysis was performed evaluating three different experiments and statistical analysis was performed by comparing NA-treated cells with control cells (*) and NA + WS433-, NA + CPZ- and NA + WS433 + CPZ- with NA-treated cells (**), p ≤ 0.01.

**Figure 6:**
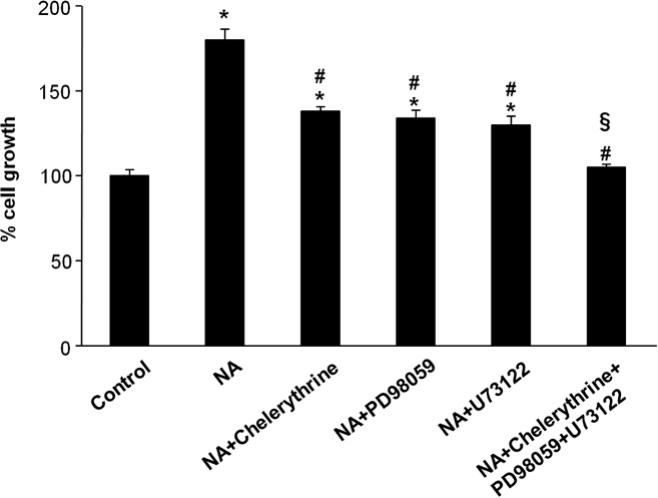
**PLC, PKC and MAPK inhibitors block the NA-induced PC3 cell survival.** Cell growth was assessed by MTT assay in PC3 cells treated for 24 h with vehicle (control) or NA (100 μM), alone or in combination with chelerythrine (0.5 μM), U73122 (5 μM) and PD98059 (50 μM). Data shown are the mean ± SD of three separate experiments**.** Statistical analysis was performed by comparing NA-treated with control cells (*), NA + chelerythrine-, NA + PD98059-, NA + U73122- and NA + chelerythrine + PD98059 + U73122- with NA-treated cells (#) and NA + chelerythrine + PD98059 + U73122- with NA + chelerythrine-, NA + PD98059- and NA + U73122-treated cells (§), p ≤ 0.01.

Then the ability of WS433 or CPZ to inhibit PLC activation, ERK1/2 phosphorylation and the production of phospho-(Ser) PKC substrates was assessed. In regard to PLC activation, when used in combination with NA CPZ, but not WS433, partially inhibited IP_3_ production; however, NA administered in combination with WS433 and CPZ induced a IP_3_ production, but at a lower rate than NA in combination with CPZ (Figure 5A). No IP_3_ production was found when using WS433 and CPZ alone respect to control PC3 cells (Additional file 2: Figure S1C). The NA-induced ERK1/2 phosphorylation was markedly affected by both the α_1D_-AR and TRPV1 antagonists or by their combination (Figure 5B), whereas the NA-induced increase of (Ser)-PKC substrate phosphorylation was reduced only by CPZ and WS433 used in combination (Figure 5C). No ERK1/2 and (Ser)-PKC substrate phosphorilation was found when using WS433 and CPZ alone respect to control PC3 cells (Additional file 2: Figure S1D, E). Next, the ability of pharmacological inhibitors of PLC, PKC and ERK to inhibit the NA-induced PC3 cell growth was evaluated. We found that U73122, chelerythrine chloride and PD98059, used at the highest doses that did not affect cell viability (Additional file 2: Figure S1F), markedly inhibited the cell growth of NA-stimulated PC3 cells; in addition, when all the three pharmacological inhibitors were used in combination, they completely reverted the NA-mediated effects (Figure 6). These results confirmed that the PLC/PKC/ERK axis plays a major role in the control of α_1D_-AR- and TRPV1-dependent PC3 cell growth.

### NA stimulates the proliferation of PC3 cells in a α_1D_-AR- and TRPV1-dependent manner

**Figure 7:**
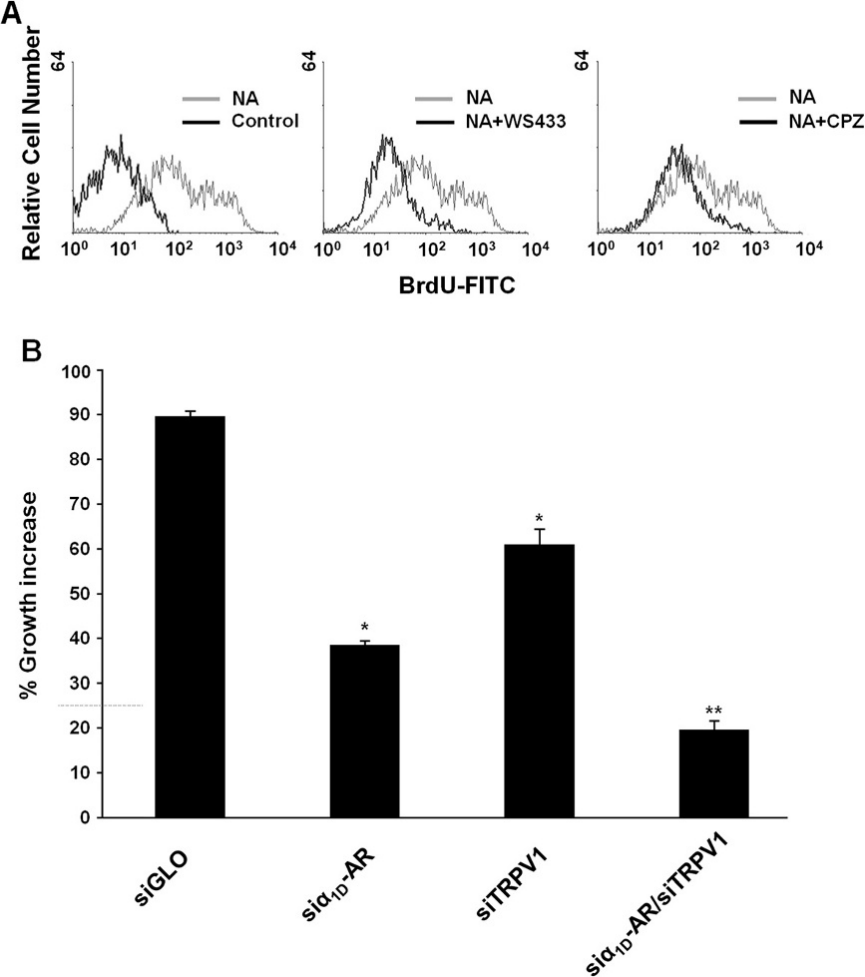
**NA stimulates the proliferation of PC3 cells in an** α_**1D**_ **-AR and TRPV1 dependent manner. (A)** The proliferation of PC3 cells was analysed by labelling with BrdU. The cells were treated with vehicle (control) or NA (100 μM) for 24 h, alone or in combination with WS433 (1 μM) and CPZ (1 μM). Cells were stained with an anti-BrdU FITC-conjugated Ab and analysed using a FACScan flow cytometer. Data shown are representative of one of three separate experiments. **(B)** siGLO-, siα_1D_-AR-, siTRPV1- and siα_1D_-AR/siTRPV1-transfected PC3 cells were treated for 24 h with NA (100 μM), and cell proliferation was assessed by BrdU labelling. The percentage increase of cell proliferation was evaluated with respect to vehicle-treated cells. The data shown are the mean ± SD of three separate experiments. Statistical analysis was performed by comparing siα_1D_-AR-, siTRPV1- with siGLO-transfected cells (*), and siα_1D_-AR/siTRPV1- with siα_1D_-AR- or siTRPV1-transfected cells (**), p ≤ 0.01.

We previously reported the role of α_1D_-AR in the NA-induced proliferation of PC3 cells [17]. Thus, we evaluated the potential contribution of TRPV1 to the NA-induced α_1D_-AR-mediated proliferation of PC3 cells. We found that WS433 and CPZ (Figure 7A) inhibited NA-induced proliferation. No change in proliferation rate was observed when using WS433 and CPZ alone respect to control PC3 cells (Additional file 2: Figure S1G). Then, to strengthen these findings, we performed cell proliferation in single (α_1D_-AR or TRPV1) and double (α_1D_-AR/TRPV1) silenced PC3 cells (Additional file 3: Figure S2A, B, C). A marked decrease in NA-induced proliferation was already evident in siα_1D_-AR and siTRPV1 cells even if a stronger inhibition (< 80%) was observed in double siα_1D_-AR/siTRPV1 PC3 cells (Figure 7B).

## Discussion

TRP channels may participate in Ca^2+^ homeostasis in PCa cells [24]. It has been hypothesised that abnormal Ca^2+^ signalling may be an essential step in the α_1_-AR-mediated increased proliferation in PCa cells [3].

Herein, we firstly demonstrated that prostate cancer cell lines co-expressed α_1D_-AR and TRPV1 proteins and these receptors co-localized mainly in the plasma membrane, perinuclear region and intracellular vesicles. Therefore, we investigated whether these receptors functionally interact each other. Previously, cross-sensitisation between TRPV1 and other members of the GPCR family, such as P2X3 and CCR1 in DRG neurons [25] and CCR1/TRPV1-transfected human embryonic kidney (HEK293) cells, respectively, have been demonstrated [26].

We also evaluated the α_1D_-AR and TRPV1 co-expression at mRNA and protein levels in advanced PCa tissues. As previously reported [6], TRPV1 mRNA expression is higher in PCa than in BPH tissues. Our data demonstrated that α_1D_-AR mRNA and protein levels were markedly increased in PCa samples compared with BPH specimens and, more importantly, that there is a great correlation between α_1D_-AR and TRPV1 expression levels. Moreover, a strong reduction of the α_1D_-AR and TRPV1 mRNAs was observed in 5/37 PCa patients who received neoadjuvant androgen deprivation therapy compared with untreated PCa patients. This result suggests that the transcriptional activity of TRPV1 and α_1D_-AR may be androgen dependent. Chromatin immunoprecipitation analysis revealed that TRPV1 is a novel androgen receptor target gene in castration-resistant C4-2 PCa cells [27]. Similarly, decreased TRPV6 levels were detected in androgen-insensitive tumours after androgen deprivation therapy [2]. No data on the relationship between α_1D_-AR and androgen receptors has been published to date; however, the expression of β_2_-AR, a well-known activator of the androgen receptors, was transiently down-regulated in hormone-sensitive LNCaP cells treated with the anti-androgen compound bicalutamide [28, 29]. Thus, there may be a similar effect on α_1D_-AR in PCa patients treated with anti-androgen drugs.

α_1D_-AR has a 10- to 100-fold higher affinity for endogenous catecholamines than the α_1A_- and α_1B_-AR subtypes [30]. Therefore, we studied whether the binding of NA to α_1D_-AR sensitises TRPV1 in PC3 cells. By protons release and Ca^2+^ flux analysis NA resulted in a rapid response, which was inhibited by WS433 and CPZ. Moreover, NA stimulates a cross-talk between α_1D_-AR and TRPV1 in PC3 cells that involves the PLC-PKC-ERK pathways. In particular, NA sensitises TRPV1, but not α_1D_-AR, to activate the PLC pathway. This result is in agreement with previous findings demonstrating a weaker coupling between PLC and α_1D_-AR than between PLC and other α_1_-AR subtypes [9, 31]. Calcium flowing through TRPV1 activates PLC, and the resulting depletion of PIP_2_ plays a role in PKC-dependent TRPV1 sensitisation [32]. Moreover, recently TRPV1 has been demonstrated to be either inhibited or activated by PIP_2_ [33]. In this regard, we found that the IP_3_ production is a TRPV1-dependent event; WS433 in combination with CPZ significantly inhibited the IP_3_ production to a greater extent than CPZ alone, suggesting a co-stimulatory effect of α_1D_-AR signalling in sustained TRPV1-dependent PLC activation. Moreover, the CPZ and WS433 drug combination significantly reduced the NA-induced phospho-(Ser) PKC substrate phosphorylation. Finally, both CPZ and WS433 antagonists alone markedly inhibited the ERK1/2 phosphorylation, although the maximal effect was evidenced by their use in combination, suggesting that ERK1/2 represents a downstream component of NA-induced TRPV1 and α_1D_-AR signalling pathway. In this regard, in human HEK293 cell line transfected with α_1D_-AR, a constitutive ERK1/2 phosphorylation, which was reduced by incubation with the selective α_1D_-AR antagonist BMY7378, was evidenced; in addition a rapid and transient ERK1/2 phosphorylation, that was not inhibited by the PKC inhibitor, Ro-8425, following α_1D_-AR activation was demonstrated [34]. Moreover, α_1D_-AR activation stimulated the ERK pathway in CHO cells [35] and in lacrimal gland epithelial cells [36].

Previous reports have indicated the contribute of α_1D_-AR in NA-induced proliferation of PC3 cells [17], however no data on the potential effects of TRPV channels in PCa proliferation were reported. Herein we found that complete abrogation of NA-induced increase in PC3 cell proliferation was reached only in double-silenced α_1D_-AR/TRPV1 PC3 cells. The silencing of the α_1D_-AR or TRPV1 gene or the use of WS433 or CPZ alone, partially but not completely, inhibited the NA-induced effects suggesting that these receptors act cooperatively.

Coupling of α_1_-AR to Ca^2+^-permeable TRPCs channels has been reported in LNCaP cells [24, 37, 38] and WB4101 and the TRP channel blockers 2-ABP and SK&F 96365 [24]. Furthermore, naftopidil, an α_1D_-AR selective antagonist, has been also reported to affect the proliferation of human prostate epithelial cells [39], and labedipinedilol-A, which shows high selectivity for α_1A_- and α_1D_-AR, to inhibit the NA-stimulated proliferation and ERK phosphorylation in LNCaP and PC3 cells [40]. Thus, because of the lack of potent and selective subtype-specific α_1_-AR antagonists, experimental or clinical trials using these compounds are few [41, 42].

## Conclusion

Overall, our findings, demonstrating a functional cooperative role played by the α_1D_-AR/TRPV1 cross-talk in NA-induced proliferation of PCa cells, strongly support for a new pharmacological approach in the care of PCa by targeting both the α_1D_-AR and TRPV1 receptors.

## Electronic supplementary material

Additional file 1: Table S1: Patient demographics. IQR = interquartile range; PSA = prostate-specific antigen. (XLSX 31 KB)

Additional file 2: Figure S1: Effects of WS433 and CPZ antagonists and chelerythrine, PD98059, U73122 inhibitors in PC3 cells. **(A)** The measurement of the extracellular acidification rate was performed as above described with PC3 cells treated for 30 sec with vehicle (control) or WS433 (1 μM) or/and CPZ (1 μM). Data shown are the mean ± SD of three separate experiments. **(B)** The time course of [Ca^2+^]_i_ increase in Fluo-3-loaded PC3 cells vehicle-treated (control) or treated with WS433 or/and CPZ was evaluated by FACS analysis. Data shown are the mean ± SD of three separate experiments. **(C)** The measurement of IP_3_ levels performed as above described in PC3 cells vehicle-treated (control) or treated for different times with WS433 (1 μM) or/and CPZ (1 μM). Data shown are the mean ± SD of three separate experiments. **(D-E)** Lysates from PC3 cells vehicle-treated (control) or treated for different times with WS433 (1 μM) or/and CPZ (1 μM) were separated on SDS-polyacrylamide gels, transferred and then blotted with anti-pERK, ERK and anti-phospho-(Ser) PKC substrate Abs. The GAPDH protein level was evaluated as a loading control. Data shown are representative of one out of three separate experiments. **(F)** Cell growth was assessed by MTT assay in PC3 cells treated for 24 h with vehicle (control) or with chelerythrine (0.05-5 μM), PD98059 (1–100 μM) and U73122 (0.5-50 μM). Data shown are the mean ± SD of three separate experiments. Statistical analysis was performed by comparing chelerythrine, PD98059 and U73122 treated cells with control (*), p ≤ 0.01. **(G)** The proliferation of PC3 cells was analysed by labelling with BrdU. The cells treated with WS433 (1 μM) or CPZ (1 μM) were stained with an anti-BrdU FITC-conjugated Ab and analysed using a FACScan flow cytometer. Data shown are representative of one of three separate experiments. (TIFF 13 MB)

Additional file 3: Figure S2: Silencing of the α_1D_-AR and TRPV1 genes in PC3 cells. **(A)** The α_1D_-AR and TRPV1 mRNA levels were evaluated by qRT-PCR in siGLO-, siα_1D_-AR- and siTRPV1-transfected PC3 cells. The relative α_1D_-AR and TRPV1 expression levels, normalised to the β-actin mRNA level, were calculated using siGLO as a calibrator. **(B)** Lysates from siGLO-, siα_1D_-AR- and siTRPV1-transfected PC3 cells were separated by SDS-polyacrylamide gel electrophoresis and probed with anti-α_1D_-AR or anti-TRPV1 Abs. The GAPDH protein level was evaluated as a loading control. Representative immunoblots are shown. **(C)** FACS analysis was performed in PC3 cells double silenced for α_1D_-AR and TRPV1 genes. Silenced cells were double-stained with anti-TRPV1 and anti-α_1D_-AR Abs followed by respective secondary Abs. Data shown are representative of one of three separate experiments. (TIFF 3 MB)

## Abbreviations

α_1_-AR: Alpha_1_-adrenergic receptor
PCa: Advanced prostate cancer
Ab: Antibody
mAb: Mouse monoclonal Ab
BPH: Benign prostate hyperplasia
BrdU: Bromodeoxyuridine
CPZ: Capsazepine
WS433: Clopenphendioxan
[Ca^2+^]_i_: Calcium influx
DAG: Diacylglycerol
DMSO: Dimethyl sulfoxide
qRT-PCR: Quantitative real time-PCR
ERK1/2: Extracellular signal-regulated kinase 1/2
ECAR: Extracellular acidification rate
FBS: Fetal bovine serum
FITC: Fluorescein isothiocyanate
GPCRs: G-protein-coupled receptors
HRP: Horseradish peroxidise
HEK293: Human embryonic kidney
PIP2: Inositol 4,5-bisphosphate
IP_3_: Inositol 1,4,5-triphosphate
I-RTX: Iodoresinferatoxin
NA: Noradrenaline
PE: Phycoerythrin
PLC: Phospholipase C
PKC: Protein kinase C
PSA: Prostate specific antigen
siTRPV1: siGENOME SMARTpools for TRPV1
siα_1D_-AR: α_1D_-AR
siGLO: siCONTROL non-targeting small interfering RNA
TRPV1: Transient receptor potential vanilloid type 1
TRPC: Transient receptor potential channel.
