# Supplementary figures and images for: Cross-talk between alpha1D-adrenoceptors and transient receptor potential vanilloid type 1 triggers prostate cancer cell proliferation
Source: BMC Cancer. 2014 Dec 7;14:921. doi: 10.1186/1471-2407-14-921 (PMC4306515; doi:10.1186/1471-2407-14-921)

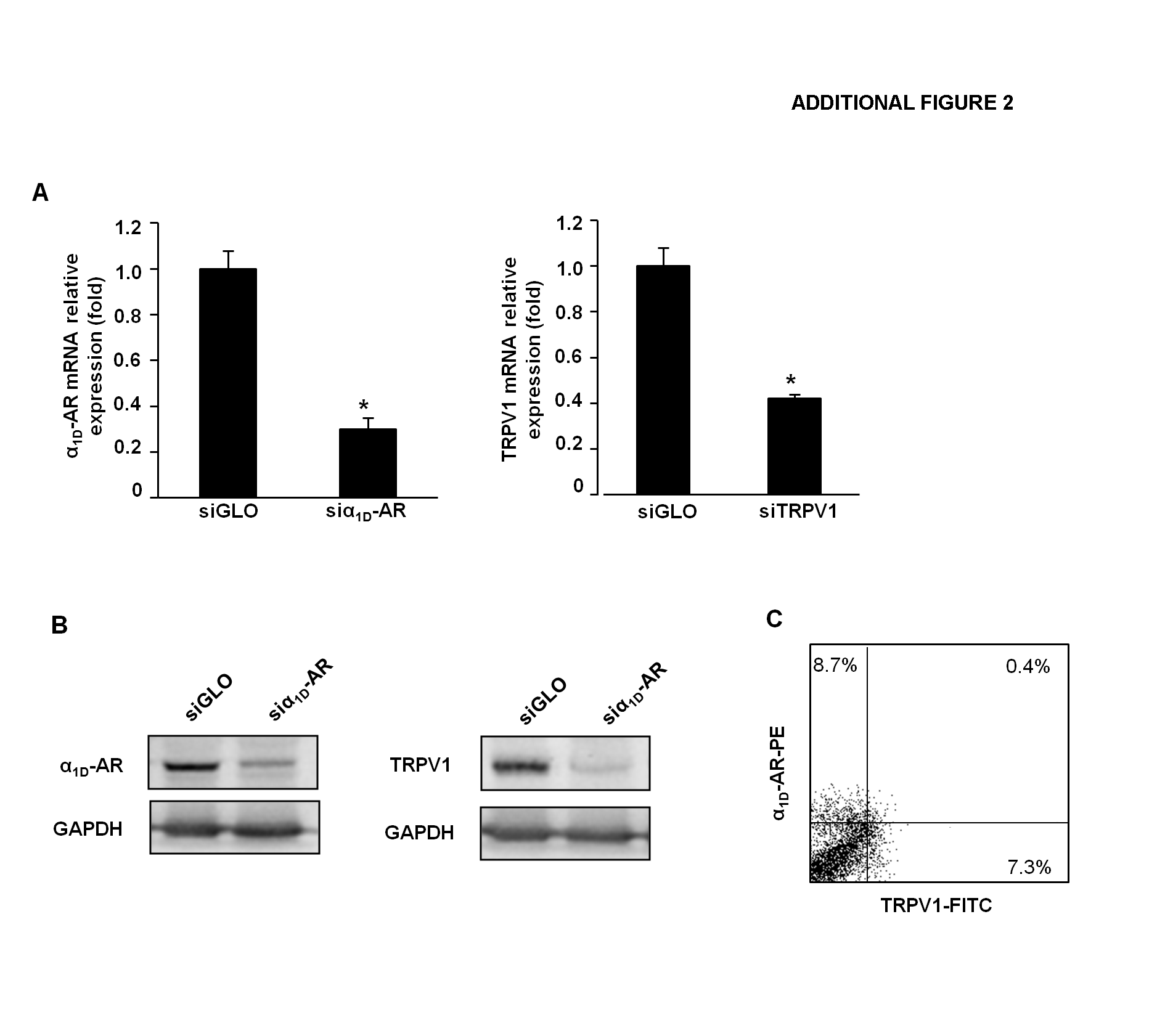

Supplement: Supplementary file 3 — Additional file 3: Figure S2: Silencing of the α1D-AR and TRPV1 genes in PC3 cells. (A) The α1D-AR and TRPV1 mRNA levels were evaluated by qRT-PCR in siGLO-, siα1D-AR- and siTRPV1-transfected PC3 cells. The relative α1D-AR and TRPV1 expression levels, normalised to the β-actin mRNA level, were calculated using siGLO as a calibrator. (B) Lysates from siGLO-, siα1D-AR- and siTRPV1-transfected PC3 cells were separated by SDS-polyacrylamide gel electrophoresis and probed with anti-α1D-AR or anti-TRPV1 Abs. The GAPDH protein level was evaluated as a loading control. Representative immunoblots are shown. (C) FACS analysis was performed in PC3 cells double silenced for α1D-AR and TRPV1 genes. Silenced cells were double-stained with anti-TRPV1 and anti-α1D-AR Abs followed by respective secondary Abs. Data shown are representative of one of three separate experiments. (TIFF 3 MB) [file 12885_2014_5096_MOESM3_ESM.tiff]
